# The therapeutic dental challenge of xeroderma pigmentosum patients: case report

**DOI:** 10.11604/pamj.2023.44.159.34950

**Published:** 2023-04-04

**Authors:** Yasmine Hanine, Sonia Ghoul, Amine Cherkaoui

**Affiliations:** 1 International University of Rabat, College of Health Sciences, International Faculty of Dental Medicine, BioMed Unit, Technopolis Parc, Rocade of Rabat-Salé, Sala-Al Jadida, Morocco,; 2 Department of Periodontology, Faculty of Dental Medicine, Mohammed V University in Rabat, Rabat, Morocco

**Keywords:** Xeroderma pigmentosum, dental care, ultraviolet rays, case report

## Abstract

Xeroderma pigmentosum (XP) is a rare genetic disease characterized by a hypersensitivity to ultraviolet (UV) radiation leading to defective deoxyribonucleic acid (DNA) repair and predisposing to skin tumorigenesis. This paper reports the safe approaches used for the dental treatment of XP patients, controlling the ultraviolet (UV) sources at the dental office. An XP 29-year-old woman was referred for oral pain and sensitivity at the service of periodontology, UV rays were checked with a UV-meter. During the examination, the patient kept her sunglasses while the practitioner was dressed in dark colors using an anti-UV filter over the surgical light. Facial dark brown pigmentations, limited mouth opening, tumor resection scar on the tongue, moderate periodontitis, and dental caries were noticed. Moderate periodontitis and dental caries were diagnosed. Treatment was planned in collaboration with the dermatologist. Soft scaling and root planning were performed in short sessions and self-curing material was used for coronary fillings after caries removal. In taking care of XP patients, particular attention should be given by dental professionals to: i) the office management for a UV-safe environment; ii) the adoption of suitable dental care and safe biomaterials with short sessions and regular controls; and iii) the adoption of personal protections by patients and practitioners.

## Introduction

Xeroderma pigmentosum (XP) is a rare autosomal recessive disorder that is characterized by photoaging and high sensitivity to ultraviolet (UV) exposure [1]. This disease is a result of an enzyme deficiency known to be useful for the repair of UV-damaged DNA. Considering that no cure is yet available, XP patients should be completely protected and isolated from all types of UV because they consequently have a high probability to develop different neurologic and skin alterations.

On another note, the UV light emitted by sunlight is not only valuable for general health, by stimulating the production of vitamin D in the skin, but it is particularly useful in the dental field, whether for the polymerization of biomaterials or for improving bonding in restorative dentistry [2] or osseointegration in implantology [3]. Given their virucidal, bactericidal, and fungicidal properties, UV was even recommended as a disinfection protocol during the COVID-19 pandemic for dental practices [4]. Consequently, several controlled and uncontrolled sources of UV may coexist at the dental office.

The aim of this paper is to describe how to take care of a xeroderma pigmentosum patient, by controlling the UV sources at the dental office and adopting a safe approach for their dental care.

## Patient and observation

**Patient information:** a 29-year-old woman was referred to the department of periodontology for oral pain and bleeding on brushing. The patient arrived wearing sunglasses and protective clothing, she declared herself suffering from xeroderma pigmentosum. The medical history revealed that the patient has already had caries treatments elsewhere.

**Figure 1:**
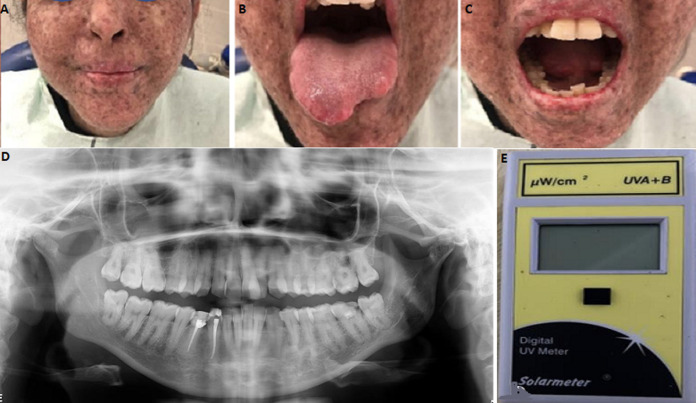
clinical examination of the XP patient: A) multiple hyper pigmented freckles and macules on the face; B) scar due to the resection of a tumour on the tongue; C) limited mouth opening; D) panoramic radiograph showing the number, shape, and position of the teeth; E) patient´s UV meter used to monitor the UV exposure

**Clinical findings:** the patient showed dark brown pigmentations all over her face (Figure 1A) and limited mouth opening (Figure 1B). On the endobuccal examination, there was a tumor resection scar on her tongue (Figure 1C).

**Timeline:** the patient was born to normal parents with consanguineous marriage, and no other family member had a similar syndrome. She was diagnosed with XP when she was 2 months old, and she had no specific neurodegenerative condition at the time of consultation.

**Diagnostic assessment:** a panoramic radiograph was performed as the limited mouth opening did not allow the use of retro alveolar radiographs (Figure 1D). A periodontal probing was performed and revealed the presence of periodontal pockets, localized to the lower incisors. The dermatologist was contacted to seek other medical information about the patient and to discuss the treatment approaches.

**Diagnosis:** the patient diagnosed with XP, presented unsatisfactory hygiene and moderate localized periodontitis complicated by dental crowding and the lack of oral hygiene causing inflammation which explains the bleeding of the gum. The patient also presented several caries, that caused dental pain.

**Therapeutic intervention:** the first appointment was short and dedicated to emergency care. Before the oral examination, the UV at the office was assessed with a UV-meter (Figure 1E), and UV exposure was checked for a safe environment. At first, patient care included protection against UV rays. Indeed, the patient was equipped with her sunscreen that she renewed every hour, and the appointment was given at the end of the afternoon. The light was kept off at the perioperative area and a UV filter was used over the surgical light while the patient kept her sunglasses on her eyes. Periodontal treatment included hygiene motivation and teaching of proper brushing methods followed by ultrasonic scaling. Decay removal was carried out by using an electrical handpiece ball burr on low speed, the filling was done using a self-curing glass ionomer restorative material (RIVA SELF CURE*). The patient was seen one week after the elimination of etiological factors and root planning was realized.

**Follow-up and outcomes:** after 2 months, a re-evaluation was performed, the pain had disappeared, the bleeding had stopped, and the depth of the pockets had decreased. A maintenance phase was initiated, to see the patient twice a year. The sessions include hygiene motivation and plaque control.

**Patient perspective:** the patient expressed her gratitude for the care she received and expressed optimism about her condition's progress.

**Informed consent:** because her condition was unusual, the patient agreed to the publication of a case incorporating her clinical data because she wished to aid in the treatment of similar cases in the future.

## Discussion

**Figure 2:**
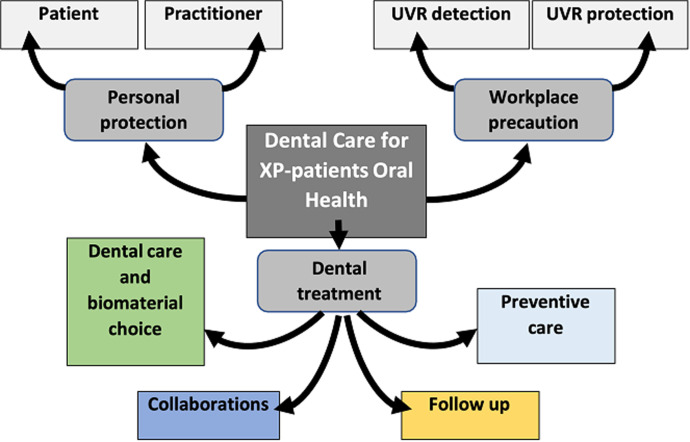
mind mapping of dental care for XP-patients

Appropriate precautions were taken for the patient, considering her dental needs, the working environment, and the dental procedures. Sources of UV were identified and avoided as much as possible. These sources included: i) external sunlight that could be reflected on walls, ceiling, and even the operator´s clothes; ii) UV light from the surgical light; iii) UV light from the flash when photographing; iv) light from the UV lamps used for tooth bleaching and; v) UV lights from the photopolymerizing lamps. Even if, the patient was aware of her health limits since a young age, she was less motivated for dental health, and education in oral hygiene was necessary. Dental care for XP patients deserves to be deeply thought of and a mind mapping approach was proposed and developed in three points (Figure 2).

**Personal protection:** the use of personal protective equipment, such as sunscreen, protective eyewear, hats, gloves, socks, or clothes designed with high UV protection factor and long pants and sleeves is recommended for XP patients. Moreover, according to Sarkar, the best colors to wear to help prevent UV radiation are dark shades [5]. Interestingly, these dark colors absorb also light and UV rays far better than light or pastel colors, avoiding their reflection. So, dark colors are recommended for both practitioners and patients not only to prevent UV exposition, but also to avoid light reflection.

**Workplace precaution:** several strategies have been proposed to reduce ultraviolet radiation exposure for outdoor workers [6,7], but little is known about strategies to reduce UV exposure indoors. UV safety is essential for XP patients, and it is important that dentists think to organize dental offices anticipating the management of these patients. The sources of UV radiation are multiple, and their detection is necessary to be controlled. So far, UV radiation could be detected in at least two ways, either using UV-beads or UV-meter. UV-beads contain pigments that turn colors when exposed to UV light. The beads are white in ordinary visible light, but different colors could appear in UV light. However, these beads give only an approximate appreciation of the UV exposure. For a quantitative appreciation of the UV, a portable and highly sensitive UV-meter is preferred as it measures the UV either in microW/ cm^2^ and/or doses microJ/cm^2^. Such a tool is recommended for XP patients not to exceed 2-30 microW/cm^2^ [8], corresponding to the weak radiation at dawn or sunset time. UV filters over lights and cameras could also be described [9], but caution should be taken given that the filters induce an uncontrolled change in the wavelength of the light and lead to instability at long exposure. Risks could also be limited by covering the rest of the patient´s body with drapes and towels during the intervention. Furthermore, at the dental office, the use of dark panels to absorb light, such as in photography studios, could be helpful.

**Dental treatment:** even if the working environment could be controlled and the UV-intensity limited to the recommended levels, the duration of the treatment session should be reduced to the minimum to manage the daily UV-cumulative value of the patient. As preventive care, the dentist is advised against the use of UV light-curing units with these patients while special attention is given to the monitoring of precancerous lesions. Due to the hypersensitivity of the skin and mucosa, irritating biomaterials and mouthwashes should be avoided during dental care, while excision of cancerous lesions should be extensive.

**Figure 3:**
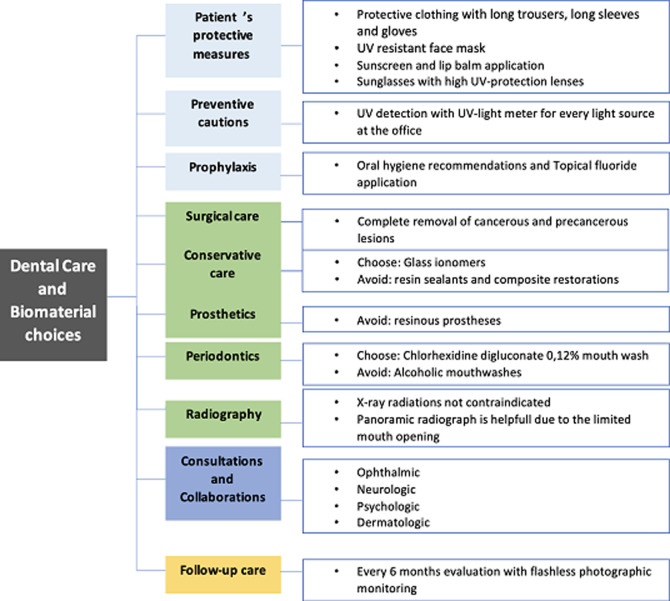
dental therapeutic precautions to be taken when dealing with a xeroderma pigmentosum patient

Oral health has been a bit neglected compared to the overall health of our patient. In fact, our patient has already undergone dozens of operations considering her young age. The role of the dentist is important to educate the patient, to prevent, treat and establish a follow-up in collaboration with other health professionals (Figure 3).

## Conclusion

Xeroderma pigmentosum is a rare and life-threatening disease with various malignant tumors that can occur at an early age. Avoiding UV-exposure and protecting against the progression of the disease are the only ways to limit the damage of the syndrome. The dentist plays an important role in giving psychological support to the family and improving the quality of life and the vital prognosis of the XP-patients. Particular attention should be given to: i) the dental office management for a UV-safe environment; ii) the adoption of suitable dental care and materials making sure that the patients are aware of the importance of oral health in collaboration with other health professionals and; iii) adoption of personal protection either for patients or practitioners.
